# Chemical profiling, in vitro antimicrobial and antioxidant activities of pomegranate, orange and banana peel-extracts against pathogenic microorganisms

**DOI:** 10.1186/s43141-021-00151-0

**Published:** 2021-05-30

**Authors:** Safynaz Magdy Hanafy, Yasser Mohamed Abd El-Shafea, Waleed Diaeddeen Saleh, Hayam Mohamed Fathy

**Affiliations:** 1 Regional Centre for Food and Feed (RCFF), Agriculture Research Center (ARC), Giza, Egypt; 2 Department of Microbiology, Faculty of Agriculture, Cairo University, Giza, Egypt

**Keywords:** Antimicrobial activity, Pomegranate peel extracts, Phytochemical assay, GC-MS analysis

## Abstract

**Background:**

The use of natural preservatives became of great interest; good examples of these natural preservation agents are plant peels. The use of plant peels has dual benefits; first is their antimicrobial activity against food-borne pathogens, while the second is minimizing agro-industrial wastes.

**Results:**

The evaluation of the antimicrobial potential of both methanolic and ethanolic extracts of three fruit peels (orange, pomegranate, and banana), against 4 Gram-positive (G^+^), 3 Gram-negative bacteria (G^−^), and 2 fungal strains revealed that both pomegranate peel extracts exhibited significantly higher inhibitory effect on all tested G^+^ bacteria*.* Methanolic extract of pomegranate peel gave higher activity than the ethanolic one against G^+^ and G^−^ bacteria except for *S. typhimurium*. Against *A. flavus* and *A. niger*, both pomegranate and orange extracts showed activity ranging between 65 and 100% more than the positive control. The ethanolic extracts of all tested peels showed a considerable capacity of antioxidant compounds compared to the methanolic extracts. The highest antioxidant capacity was found for ethanolic and methanolic extracts of pomegranate, 66.870 and 56.262 mg/ml, respectively. Generally, the concentration of total phenolic compounds was higher than that of total flavonoids followed by tannins. The highest readings of all tested constituents were reported for pomegranate extracts followed by orange and then banana. The total phenolic content, total flavonoids, and tannins were proportional to antioxidant values. GC-MS of pomegranate peel extracts identified 23 compounds in the methanolic extract versus 31 compounds in the ethanolic one. These components were identified based on their retention times and mass spectral fragmentation pattern. 5-hydroxymethylfufural (HMF) represented the major component in both methanolic and ethanolic extracts with peak area percentage of 65.78% and 48.43%, respectively.

**Conclusions:**

The results showed negative effect of methanolic and ethanolic extracts of pomegranate on G^+^ and G^−^ bacteria and two fungal pathogenic strains. The phytochemical analysis regarded these results to the high content of phenols, flavonoids, and tannins. GC-MS chromatogram identified many compounds known to be effective as antioxidants and antibacterial and antifungal agents. These indications show that pomegranate peel may be a superior natural food-preserver, but further studies about the suitable formulation, dosage, and possible side-effects are still needed.

## Background

Food-borne illness due to consumption of food contaminated with pathogenic bacteria and/or their toxins is a vital concern to public health. The symptoms of such illness range from vomiting, nausea, and diarrhea to long-term diseases such as liver or kidney failure, cancer, and neural or brain disorders [1]. The most common bacteria causing food-borne illness are *Escherichia coli*, *Staphylococcus aureus*, *Salmonella typhimurium*, *Listeria monocytogenes*, *Clostridium botulinum*, *Bacillus cereus*, *Vibrio parahaemolyticus,* and others. Food poisoning is still a concern for both consumers and the food industry despite the use of various preservation methods. Food processors, food safety researchers, and regulatory agencies are continuously concerned with the high and growing number of illness outbreaks caused by some pathogenic and spoilage microorganisms in foods [1, 2].

In food industry, addition of synthetic or chemical agents like formaldehyde, sulfites, nitrates, sorbates, and benzoates and synthetic antioxidants are most widely used as preservatives. Although the use of such chemicals is efficient, it may cause severe side-effects due to their accumulation in the food chain that may affect human health. Thus, the food industry has aimed to move toward the ways of clean labeling and eliminate synthetic preservatives from food formulations and alternate them with natural preservatives [3–8].

In light of the evidence of the rapid prevalence of multi-drug-resistant isolates, the need to discover new antimicrobial agents is of excessive importance. Many plants have been used because of their antimicrobial activities against pathogenic microorganisms, which are due to phytochemicals synthesized in the secondary metabolism of the plant such as flavonoids, saponins, tannins, phenolic compounds, alkaloids, anthraquinones, glycosides, and reducing sugars [8–11].

Various studies conducted on fruit and vegetable peels revealed the presence of important constituents, which can be used for pharmacological or pharmaceutical purposes. Researchers extracted many components such as phenols, tannins, flavonoids, alkaloids, and saponins that have antimicrobial, antioxidant, and anti-inflammatory activities [8, 12, 13].

For centuries, orange (*Citrus sinensis*) peel is being used as a conventional and folkloric drug against a lot of illnesses like cancer, stomach ache, immune system diseases, diuretic, cold, vitamin deficiencies, and digestive system diseases as well as bacterial and viral infections [14, 15]. Millions of tons of orange are produced globally, of which about 20% are used as beverage in addition to sauces and dressings. The remaining peels are regarded as waste although the peel extracts of citrus are considered an origin of antioxidants, phenols, flavonoids, and antimicrobial agents [16, 17].

Gyawali and Ibrahim [18] reported that 30% of the total banana production is disposed as wastes (peels). Banana (*Musa acuminata*) peels can be applied in various industries such as cosmetics, pulp, biosorbent, biofuel, organic fertilizers, paper, and environmental cleanup and can be used as conventional and inherent drug for healing many diseases [19–21].

Pomegranate (*Punica granatum* L.) fruit has been reported to exert a promising preventive activity against several inflammatory and chronic diseases. The main composition of pomegranate by-products as well as their potential to enhance specific functionalities in food applications has recently been reviewed by [22–24] who added pomegranate seed juice by-product (PSP) as reinforcing and antimicrobial agent to fish gelatin (FG) films as a promising eco-friendly active material for food packaging applications. Also, [25] stated that pomegranate has antifungal, insecticidal, antibacterial, anticoccidial, and molluscicidal effect against both plant and human pathogens.

The present study investigates the antimicrobial and antioxidant activities of local orange (*Citrus sinensis*, var. common Balady), pomegranate (*Punica granatum*, var. Wonderful), and banana (*Musa acuminate, var. Grand nan*) peel extracts against nine pathogenic microbial strains. Furthermore, this research also aims to study the phytochemical composition of the peel extracts as well as to determine the bioactive components of the most promising extracts.

## Methods

### Plant material and extraction

Three fresh fruits belonging to different families were purchased from the Egyptian local market. These fruits were orange (*Citrus sinensis*, var. common Balady), pomegranate (*Punica granatum*, var. Wonderful), and banana (*Musa acuminate, var. Grand nan*). The fruit taxonomic identities were confirmed by the Department of Pomology, Faculty of Agriculture. The fruits were washed with running tap water followed by sterilized distilled water and then peeled. The fruit peels were dried in the oven at 40 °C for 24 h [26]. The dried peels of all fruits were ground to fine powder and stored at 4 °C.

Ethanol and methanol were separately used to extract the bioactive compounds from the three fruit peels. One hundred grams of each powdered peel was extracted with 900 ml of each solvent separately and shaked for 3 days at room temperature [27]. The extracts were filtered using Whatman No. 1 filter paper. The filtrates were concentrated by evaporating the solvents using a rotary evaporator [28]. Each dry film was dissolved in 10% DMSO, filter-sterilized (0.45 μm), stored in dark bottles, and refrigerated at 4 °C for further use.

### Test microorganisms and microbial suspensions

Nine microorganisms were used to test the antimicrobial activity of peel extracts. These test microorganisms included 4 Gram-positive bacteria, i.e., *Bacillus cereus* ATCC 33018, *Staphylococcus aureus* ATCC 25923, *Staphylococcus aureus* (MRSA) *ATCC* 43300, and *Listeria monocytogenes* ATCC 7644, along with 3 Gram-negative bacteria, i.e., *Pseudomonas aeruginosa* ATCC 9027, *Escherichia coli* ATCC 35218, and *Salmonella typhi* ATCC 14028, whereas the two tested fungal strains were *Aspergillus niger* ATCC 16888 and *Aspergillus flavus* ATCC 16883*.*

All test microbes were inoculated in Mueller-Hinton agar [29] and incubated at their optimum temperatures for 24–48h. A loopful of each tested strain was sub-cultured into 5 ml Mueller-Hinton broth medium and then incubated for 18–24 h to be used for the antimicrobial studies.

### Antimicrobial studies

According to [30], well diffusion technique was used to evaluate the antimicrobial activity of each fruit peel extract against the tested pathogenic strains. Melted Mueller-Hinton agar (MHA) was inoculated with an 18–24-h-old broth culture of each tested strain and poured into Petri plates then kept to solidify. A sterile borer was used to make 8-mm wells in each Petri plate. The wells were loaded with 50 μl of each extract, separately. Wells of the negative control plates were loaded with DMSO, while the positive control was represented by antibiotic disks. Ampicillin was used against Gram-negative bacteria, kanamycin was used against Gram-positive bacteria, and Nystatin was used against fungi. All treatments were conducted in triplicates and all plates were incubated for 24–48 h at 30–37 °C. After incubation, the zones of inhibition were recorded.

For quantification, plant extracts that gave positive results for the well diffusion assay were used to determine their minimum inhibitory concentration (MIC) following the same assessment method and medium. MIC is the lowest concentration of the tested extracts that gave zone of inhibition against pathogenic strains. Different concentrations of pomegranate peel extract were prepared as 1.25, 2.5, 5, and 10% w/v, while for both banana and orange peel extracts were as follows: 10, 20, and 30% w/v. The zones of inhibition were recorded for the different concentrations [28].

### Total antioxidant capacity assay

The total antioxidant capacity of the methanolic and ethanolic extracts was evaluated by the phosphomolybdenum method according to [31]. 0.3 ml extract was combined with 3 ml of reagent solution (0.6 M sulfuric acid, 28 mM sodium phosphate, and 4 mM ammonium molybdate). The tubes were capped and incubated in a thermal block at 95 °C for 90 min. After cooling to room temperature, the absorbance of the reaction mixture was measured at 695 nm using a spectrophotometer (Specor D250 plus-Analytik Jena) against a blank of methanol or ethanol. Total antioxidant capacity was expressed in ascorbic acid equivalent per gram dry extract.

### Phytochemical assay

Three classes of phytochemicals (tannins, phenolic compound, and flavonoids) were traced in the used peel extracts. These determinations were conducted at The Regional Centre for Food and Feed of The Agricultural Research Centre by standard qualitative methods described by [32–34]. All methods were optimized with a positive control.

### Gas chromatography-mass spectrometry (GC-MS) analysis

The chemical composition of the most potent ethanolic and methanolic peel extracts, i.e., pomegranate, was determined according to [35], using Trace GC1310-ISQ mass spectrometer (Thermo Scientific, Austin, TX, USA). The mass spectrometer had a direct capillary column TG–5MS (30 m × 0.25 mm × 0.25 μm film thickness). Helium was used as a carrier gas at a constant flow rate of 1 ml/min. EI mass spectra were collected at 70 eV ionization voltages over the range of m/z 50–500 in full scan mode. The ion source temperature was set at 200 °C. The components were identified by comparison of their retention times and mass spectra with those of WILEY 09 and NIST 11 mass spectral database.

### Statistical analysis

All determinations were conducted in triplicates and were treated by two-way analysis of variance (ANOVA); the mean values were compared by LSD (*P* ≤ 0.01) using IBM SPSS, ver. 20.

## Results

### Antimicrobial activity

**Fig. 1:**
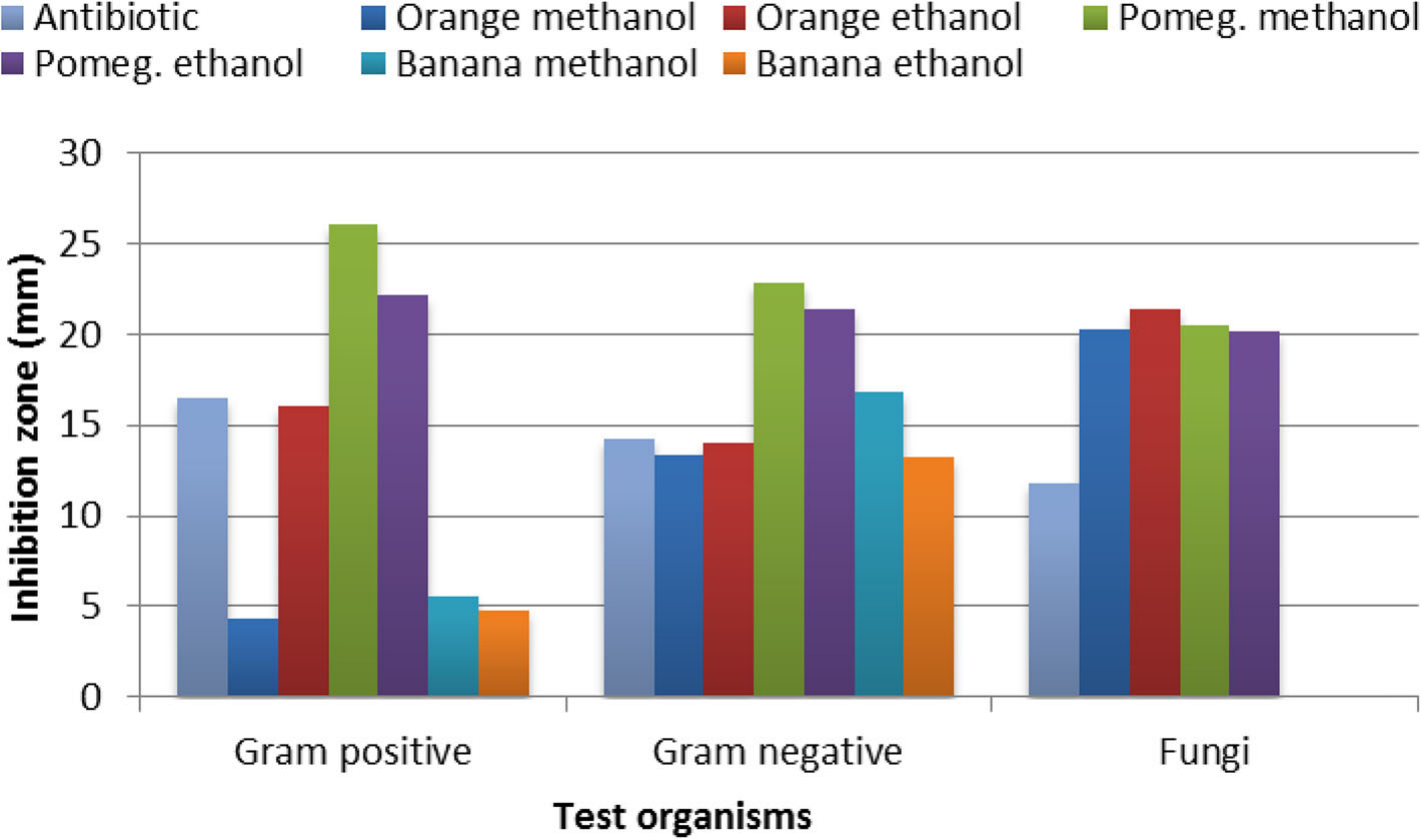
Antimicrobial activities of the used fruit peel extracts

The inhibitory effect of ethanolic and methanolic extracts of the citrus and pomegranates as well as banana peels were estimated against four Gram-positive (G^+^) and three Gram-negative bacteria (G^−^) pathogenic bacteria along with two pathogenic fungal strains. DMSO was used as negative control, while positive controls were kanamycin, ampicillin, and nystatin, in that order. The overall results are illustrated in Fig. 1.

#### Gram-positive bacteria

The antimicrobial tests of the three used fruit peels against *B. cereus, S. aureus* MRSA*, S. aureus,* and *L. monocytogenes* revealed that both pomegranate peel extracts exhibited the most significant inhibitory effect on the tested strains compared to both the treatments and the positive control, except for kanamycin against *B. cereus* in the case of ethanolic extract (Table 1).

**Table 1:** Inhibition zones of both methanolic and ethanolic extracts of the used fruit peels against some G^+^ food pathogens

Strain	Fruit peel						
	Orange^**a**^		Pomegranate^**a**^		Banana^**a**^		(+)^b^Cont.
	M	E	M	E	M	E	
***B. cereus***	17.3 ± 0.1	16.0 ± 0.1	24.3 ± 0.1	21.0 ± 0.1	-	-	21.1
***S. aureus***	-	-	26.3 ± 0.1	24.0 ± 0.3	-	-	20.0
***S. aureus (MRSA)***	-	-	27.3 ± 0.2	24.0 ± 0.2	22 ± 0.3	19 ± 0.2	14.0
***L. monocytogenes***	-	-	26.3 ± 0.1	19.7 ± 0.1	-	-	11.0
**LSD**_**0.01**_ **= 1.741; CV = 6.941%**							

Results are the means ± standard deviation

^a^Zone of inhibition in millimeters

*M* methanol, *E* ethanol, *(+)*^b^*Cont.* positive control

Methanolic extract of pomegranate peel gave the highest activity against all tested stains especially *S. aureus* (MRSA) and *S. aureus.* In the same direction, but with a little less potency, ethanolic extract of pomegranate peel showed antibacterial activity against all tested strains with special reference to *S. aureus*. *Bacillus cereus* showed sensitivity against both methanolic and ethanolic orange peel extracts while both banana peel extracts significantly affected *S. aureus* (MRSA).

#### Gram-negative bacteria

Again, the methanolic extract of pomegranate peel gave the highest antibacterial activity against Gram-negative bacteria (20.3–25.0 mm) compared to Ampicillin (12.0–18.3 mm). *Pseudomonas aeruginosa* was found to be the most sensitive tested bacterium. Pomegranate peel ethanolic extract showed a significant inhibitory effect against *S. typhimurium* (25 mm) followed by both its methanolic extract and the ethanolic extract of orange peels with equal inhibition zones of 23.3 mm. The ethanol extract of banana peels and methanolic extract of pomegranate peels gave similar antibacterial effect against *E. coli* (20.3mm) and still, significantly, higher than control (Table 2).

**Table 2:** Inhibition zones of both methanolic and ethanolic extracts of the used fruit peels against some G^−^ food pathogens

Strain	Fruit peel						
	Orange^a^		Pomegranate^a^		Banana^a^		(+)^b^Cont.
	M	E	M	E	M	E	
***S. typhimurium***	20.3 ± 0.1	23.3 ± 0.2	23.3 ± 0.2	25.0 ± 0.1	19.3 ± 0.1	19.3 ± 0.1	18.3
***E. coli***	19.7 ± 0.1	18.7 ± 0.1	20.3 ± 0.1	18.3 ± 0.2	13.0 ± 0.2	20.3 ± 0.1	12.0
***P. aeruginosa***	-	-	25.0 ± 0.1	21.0 ± 0.1	18.0 ± 0.3	-	12.6
**LSD**_**0.01**_ **= 1.686; CV = 6.173%**							

Results are the means ± standard deviation

^a^Zone of inhibition in millimeters

*M* methanol, *E* ethanol, *(+)*^b^*Cont.* positive control

Compared to the positive control, both banana peel extracts did not show significant effects against *S. typhimurium*. For *E. coli*, results reflected that banana peel ethanol extract had a significant inhibitory effect (20.3 mm) similar to pomegranate methanolic extract, whereas the methanolic extract of banana peel showed inhibitory effect against *P. aeruginosa*, significantly higher than the positive control, but still significantly lower than both the methanolic and ethanolic extracts of pomegranate peel. Many pathogens were not affected by either methanolic or ethanolic extracts of orange or banana peels.

#### Fungal strains

The extracts of fruit peels gave promising results against both fungal strains. In this study, *A. niger* and *A. flavus* were inhibited by orange and pomegranate peel extracts. Data in Table 3 show that, in comparison with positive control, *A. niger* was significantly affected by both ethanolic and methanolic extracts of orange peel followed by methanolic and ethanolic extracts of pomegranate, respectively; their inhibition zones ranged from 22.7 to 20.0 mm. On the other hand, the orange and pomegranates peel extracts had almost the same inhibitory effect against *A. flavus* which was about 65% more than the positive control.

**Table 3:** Inhibition zones of both methanolic and ethanolic extracts of the used fruit peels against some fungal food pathogens

Strain	Fruit peel						
	Orange^a^		Pomegranate^a^		Banana^a^		(+)^b^Cont.
	M	E	M	E	M	E	
*A. niger*	22.3 ± 0.2	22.7 ± 0.1	21.0 ± 0.2	20.0 ± 0.1	-	-	10.6
*A. flavus*	18.3 ± 0.2	20.0 ± 0.1	20.0 ± 0.2	20.3 ± 0.3	-	-	13.0
**LSD**_**0.01**_ **= 1.485; CV = 6.585%**							

Results are the means ± standard deviation

^a^Zone of inhibition in millimeters

*M* methanol, *E* ethanol, *(+)*^b^*Cont.* positive control

These results revealed that there was no antifungal activity of banana extracts against both fungal strains.

#### Minimum inhibitory concentration (MIC)

**Fig. 2:**
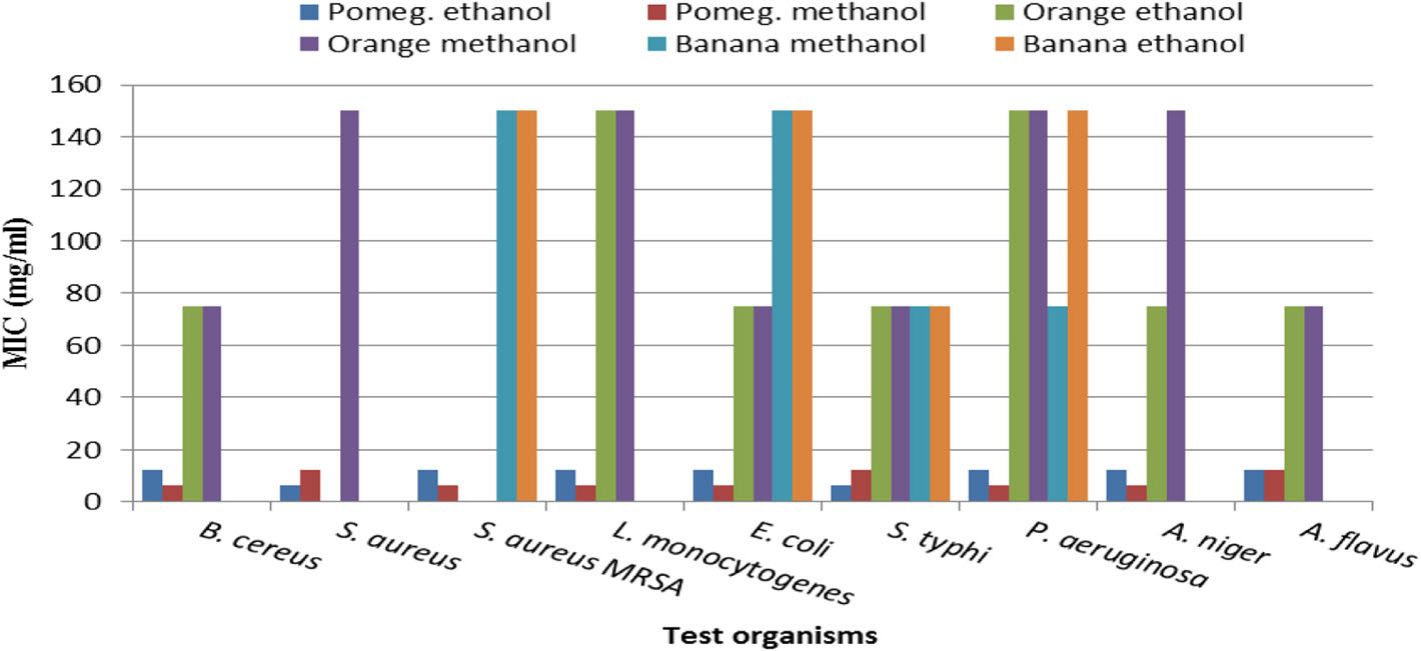
MIC values of methanolic and ethanolic extracts of pomegranate, orange, and banana peels against tested pathogens

For a quantitative view, the MIC of all used peel extracts against all tested microbes is illustrated in Fig. 2. Inhibition zones and MIC values are known for determination of antimicrobial activity.

The MIC values of pomegranate peel extracts ranged between 6.25 and 12.5 mg/ml against all tested organisms, while orange and banana peel extracts showed higher values of MIC than pomegranate peel extracts against all tested microbes (75 to 300 mg/ml). Figure 2 shows that the least value of MIC (6.25 mg/ml) was given by methanolic pomegranate peel extract on all tested microbes except for *S. aureus*, *Salmonella typhimurium,* and *Aspergillus flavus* to which it was 12.5 mg/ml. For the ethanolic pomegranate peel extract, the least MIC values (6.25 mg/ml) were achieved only versus *S. aureus* and *Salmonella typhimurium*, whereas it was 12.5 mg/ml versus all the other tested microorganisms. These results indicated the superiority of pomegranate peel in both methanolic and ethanolic extracts.

### Total antioxidant capacity

The assay depended on the reduction of Mo(VI) to Mo(V) by the antioxidant compounds in samples and formation of a green phosphate/Mo(V) complex. Table 4 presents the total antioxidant capacity of the different extracts of the used fruit peels. The ethanol extracts of the different fruit peels showed a considerable capacity of antioxidant compounds compared to the methanolic extracts.

**Table 4:** Total antioxidant capacity of the used fruit peel extracts

Fruit	Extracts (mg AAE/g)	
	Ethanolic	Methanolic
**Pomegranate**	66.87 ± 0.05	56.26 ± 0.04
**Orange**	41.23 ± 0.07	38.97 ± 0.06
**Banana**	23.86 ± 0.05	21.86 ± 0.05

*mg AAE/g* milligram ascorbic acid equivalent/gram (dry weight)

The highest antioxidant capacity was found for ethanolic and methanolic extracts of pomegranate, 66.870 and 56.262 mg AAE/g, respectively. Both orange peel extracts showing higher antioxidant levels (41.23 and 38.97 mg AAE/g) respectively than those of banana peel extracts.

### Phytochemical assessment

**Fig. 3:**
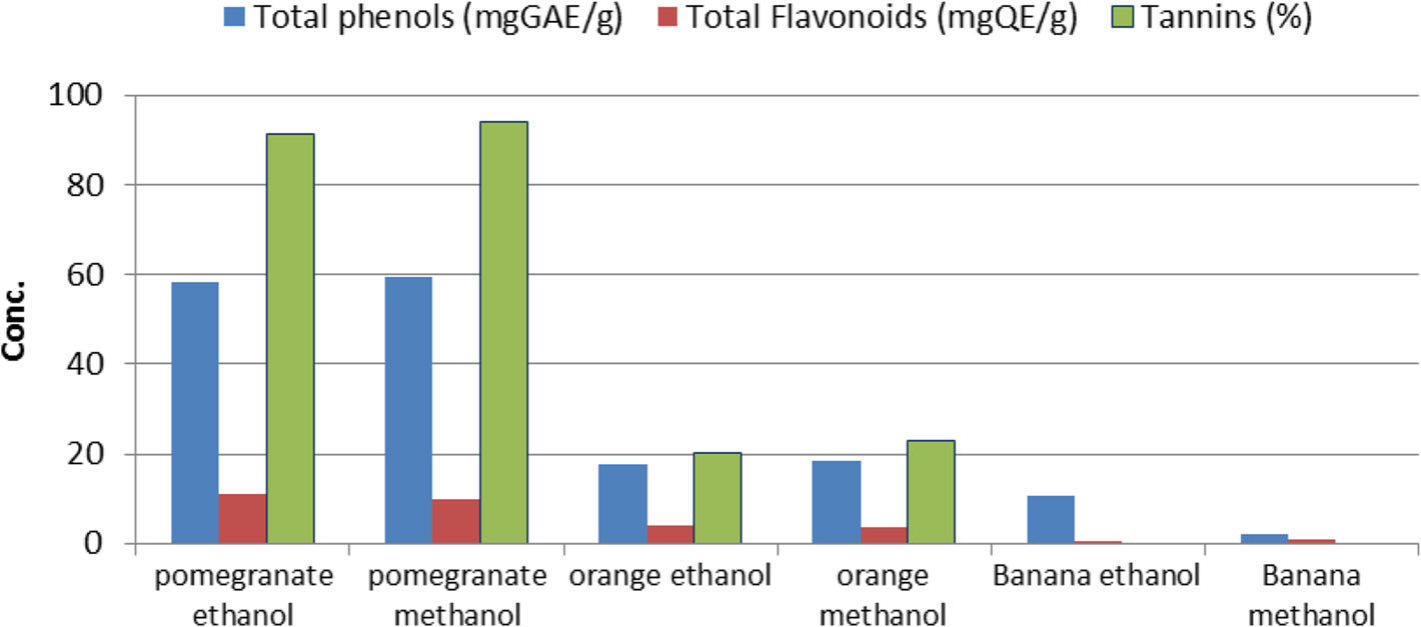
Phytochemical compounds of the plant extracts

Out of the three phytochemicals tested, phenolic compounds and total flavonoids were present in all plant extracts, while tannins were found in only four fruit extracts (Fig. 3). The highest readings of all three tested constituents were reported for pomegranate extracts, followed by orange and then banana. In all plant extracts, the concentration of total phenolic compounds was higher than the concentration of total flavonoids followed by tannins.

It is observed that extraction of the bioactive compounds from pomegranate using methanolic was more efficient than using ethanolic.

### GC-MS analysis of pomegranate peel extracts

**Fig. 4:**
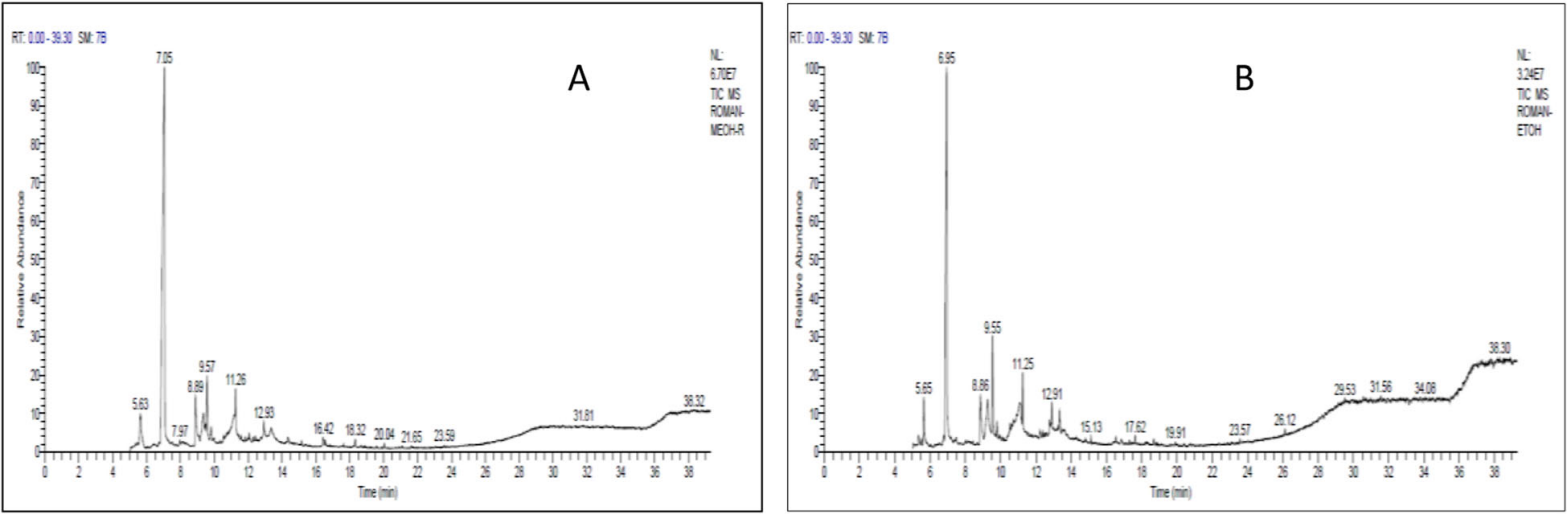
GC-MS chromatogram of methanolic (**a**) and ethanolic (**b**) extracts of pomegranate peels

As the previous results indicated the superiority of pomegranate peel extracts, they were subjected to qualitative analysis by GC-MS. The chromatograms, in Fig. 4, showed that 23 compounds were identified in the methanolic extract versus 31 compounds in the ethanolic one. These components were identified qualitatively based on their retention times and mass spectral fragmentation pattern.

The results (Table 5) reflect that 5-hydroxymethylfufural (HMF) represented the major component in both methanolic and ethanolic extracts with peak area percentage of 65.78% and 48.43%, respectively. Trioxsalen was recorded with peak area percentage of 4.87% and 7.52% in methanolic and ethanolic extracts, respectively. Also, 4H-Pyran-4-one, 2, 3-dihydro-3, 5-dihydroxy-6-methyl- was identified in both the methanolic extract (4.17%) and the ethanolic extract (4.55%).

**Table 5:** The most abundant chemical compounds in extracts of pomegranate peels by GC-MS analysis

Active compound	RT	Area%	M.F	M.W
**Pomegranate peel methanolic extract**				
4H-Pyran-4-one,2,3-dihydro-3,5-dihydroxy-6-methyl	5.63	4.17	C_6_H_8_O_4_	144
5-Hydroxymethylfurfural	7.04	65.78	C_6_H_6_O_3_	126
1,2,3,8,9,11a-hexahydro-3a,8-m ethano-9-methyl-3ah-cyclopentacyclodecene	8.89	4.79	C_15_H_20_	200
Pyrrolidine, 1-(1,6-dioxooctadecyl)-	9.35	3.44	C_22_H_41_NO_2_	351
Trioxsalen	9.57	4.87	C_14_H_12_O_3_	228
1,4-benzenediol,2-(1,1-dimethylethyl)-5-(2-propenyl)-	11.26	5.28	C_13_H_18_O_2_	206
**Pomegranate peel ethanolic extract**				
4H-Pyran-4-one,2,3-dihydro-3,5-dihydroxy-6-methyl-	5.65	4.55	C_6_H_8_O_4_	144
5-Hydroxymethylfurfural	6.95	48.34	C_6_H_6_O_3_	126
Malononitrile,(2-hydroxy-3-methoxybenzylidene)-	8.86	6.69	C_11_H_8_N_2_O_2_	200
Octadecanoic acid, 2-propenyl ester	9.27	6.51	C_21_H_40_O_2_	324
Trioxsalen	9.55	7.52	C_14_H_12_O_3_	228
Xanthosine	11.10	4.06	C_10_H_12_N_4_O_6_	284
3,4-Dihydro-2h-1,5-(3"-t-butyl)benzodioxepine	11.25	3.31	C_13_H_18_O_2_	206
à-D-glucopyranoside, methyl 2-(acetylamino)-2-deoxy-3-o-(trimethylsilyl)-,cyclic methylboronate	12.91	2.42	C_13_H_26_BNO_6_Si	331
á-D-Galactopyranoside, methyl 2,6-bis-O-(trimethylsilyl)-, cyclic methylboronate	13.33	2.09	C_14_H_31_BO_6_Si_2_	362

## Discussion

The antimicrobial effect of ethanolic and methanolic pomegranate peel extracts reported in this study was previously reported by several authors [28, 36–39]. With respect to G^+^ bacteria, our study is in line with others that reported the superiority of the methanolic pomegranate peel extract over the ethanolic one [36–39].

Concerning G^−^ bacteria, previous studies reported the activity of methanolic, ethanolic, and aqueous extract of pomegranate peel, with even better results than these of the standard antibiotic [28, 38–42].

Compared to pomegranate ethanol extract, the methanolic extract showed the greatest antimicrobial activities against different pathogens [43, 44]. Also, [45] recorded that the ethanol extract of *Musa sapientum* peel can be used to control infections caused by *Saalmonella typhi* and *E. coli*, our study showed that many pathogens were not affected by neither methanolic nor ethanolic extracts of orange or banana peels. Saleem and Saeed and Al Laham et al. [13, 46] stated that the resistance of Gram-negative bacteria against antibacterial substances may be due to outer phospholipid-membrane carrying the structural lipopolysaccharide components, which make it impermeable to lipophilic solutes, and porins constitute a selective barrier to the hydrophilic solutes.

Contrary to our study, [47] studied the antifungal activity of banana peel extracts against *A. niger*, *A. flavus,* and *Penicillium*. They found that the extract of dried banana peel powder exhibited antifungal activity against *A. niger*. The results in this study were in consistency with researchers who reported the significant inhibitory effect of *C. sinensis* and *Punica granatum L* peel extract on the growth of *F. oxysporum, P. citrinum*, *A. niger*, *F. verticillioides,* and *A. flavus* [13, 48, 49].

The antimicrobial effect of the fruits peels extracts in our study may be attributed to the presence of antimicrobial compounds in the plants such as antioxidants, phenols, flavonoids, tannins, and also the presence of secondary metabolites. Therefore, the antimicrobial activities differ from one plant extract to another due to the variance mode of action beyond their chemical composition [14, 50]. There are a lot of factors that can influence the antimicrobial activity of different fruit peel extracts. These factors such as the freshness of the used peels, the extraction method and the solvent, the country where the used plant was grown, and the time when the plant was cultivated [4].

With respect to total antioxidant capacity, the present study was in line with the findings of [45] but contradicts with the results of [51] which indicated that methanolic extract gave better results than ethanolic one using banana peels. The results of [52] indicated that the solvent used for extraction may have different impact on the antioxidant capacity of the extracts.

The superior antioxidant capacity found, in this study, for ethanol and methanolic extract of pomegranate may be regarded to the presence of total tannins and purified constituents as reported earlier by [53, 54]. Our results are in line with those of [55] who examined the antioxidant activity of nine different pomegranate peels and pulp. They found that the antioxidant activity of pomegranate peel extract was 10 times higher than the pulp extract. The results of [56] demonstrated that the pomegranate pericarp of Shahvar cultivar, which represented the highest phenolic compounds, showed the greatest antioxidant activity. In the same direction, [57] reported the higher antioxidant capacity of pomegranate peel extract compared to its seeds.

Both orange peel extracts showing higher antioxidant levels than those of banana peel extracts in this study match which with the findings of [53]. The antioxidant activity of orange peels may be regarded to glycosylated flavonoids such as hespridin and naringin [58]. The antioxidant activity of orange peel extracts was reported by [53] and regarded to the presence of various compounds, e.g., gallocatechin [59] and dopamine [60]. Wolfe et al. [61] attributed the differences in the antioxidant activities among the fruits to their differences in phenolic contents and compositions and to other non-phenolic antioxidants present in the samples.

In phytochemical assay, our study matches with [56] who reported that the methanol extract of different varieties of pomegranate had the highest phenolic content. The total phenolic content, total flavonoids, and tannins showed a proportional relationship with antioxidant values, as already observed by other authors [62]. The total phenolic content of methanolic and ethanolic extract of orange peels in this study (18.61 and 17.704 mg GAE/ml) was higher than that of [51] for the same extract (12.5 and 10.25mg GAE/ml). The extracts of banana peels were found to contain both phenolic compounds and flavonoids which is in line with the study of [45]. The present determinations did not show tannins in banana peel extracts contradicting the results of the same previously mentioned authors.

For GC-MS analysis, [63] mentioned that 5-hydroxymethylfufural has antioxidant activity while 4H-Pyran-4-one,2,3-dihydro-3,5-dihydroxy-6-methyl- has antioxidant, antimicrobial, laxative, and anticancer activities. In the same direction, the results of [64] showed bactericidal activity of HMF against a broad spectrum of bacteria with variable degrees. The antifungal activity of the pomegranate extracts is probably regarded to 4H-Pyran-4-one, 2, 3-dihydro-3, 5-dihydroxy-6-methyl compound as previously reported [65].

For the ethanolic extract of pomegranate peel components, [66, 67] reported antibacterial and antifungal activities of malononitrile while the antibacterial activity of Octadecanoic acid, 2-propenyl ester was reported by [68]. Pyrrolidine, a component of the methanolic extract of pomegranate peels in this study, was found to bear antibacterial and antifungal activities [69, 70].

## Conclusion

The present study is reporting the antimicrobial potential of three fruit peel extracts to benefit from their activity as natural preservatives. The results demonstrated the effect of methanolic and ethanolic peel extracts of pomegranate against Gram-positive, Gram-negative, and two fungal pathogenic strains. The phytochemical analysis confirmed these results by finding high content of phenols, flavonoids, and tannins in these extracts. The GC-MS chromatograms identified many compounds that are known to be effective as antioxidant, antibacterial, and antifungal agents. The indications clearly show that pomegranate peel may be a superior natural food-preserver, but further studies about the suitable formulation, dosage, and possible side-effects are still needed.

## Data Availability

The data from the study is publicly available.

## Abbreviations

G^+^: Gram-positive bacteria
G^−^: Gram-negative bacteria
ATCC: American type culture collection
MRSA: Methicillin-resistant *Staphylococcus aureus*
MIC: Minimal inhibitory concentration
GC-MS: Gas chromatography-mass spectrometry
